# Supplementary material for: Stabilized dengue virus 2 envelope subunit vaccine redirects the neutralizing antibody response to all E-domains
Source: J Virol. 2025 Apr 16;99(5):e00229-25. doi: 10.1128/jvi.00229-25 (PMC12090738; doi:10.1128/jvi.00229-25)
Supplement: Supplemental material — Table S1, list of DENV2 rE used; Fig. S1, monoclonal antibody control. [file jvi.00229-25-s0001.pdf]

Supplementary Table 1. DENV2 Soluble E proteins (Name and mutations)used as vaccines and/or in immune assays. Structures based on PDB 1OAN.

	WT	Monomer/ M2P4	SD	SD*FL
Mutations		M2 (G258E), P4 (S29K, T33V, A35M)	I2 (A259W, T262R), U6 (F279W, T280P)	I2, U6, I8 (G106D)
Previously published name[10]		SC.25	SC.14	SC.10
	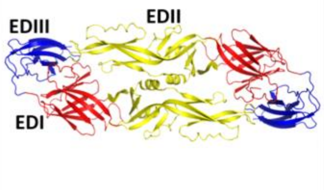	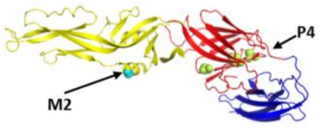	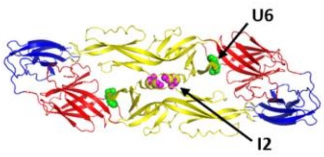	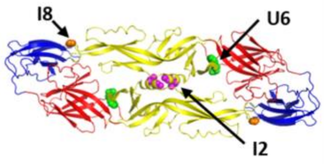

Supplementary Figure 1

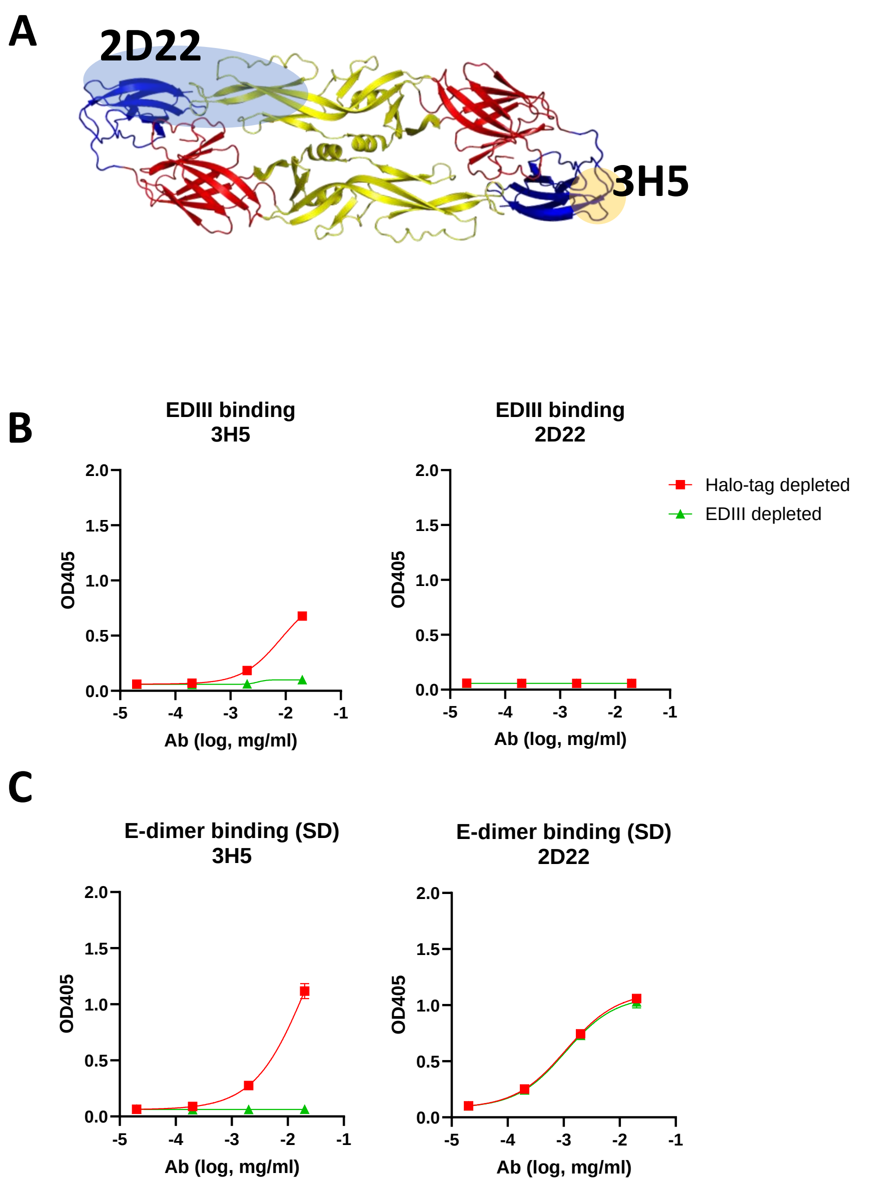

Supplementary Figure 1. Recombinant EDIII antigen depletes Abs binding to simple EDIII epitopes but not Abs binding to quaternary structure epitopes that include EDIII (**A**) Epitope location of simple EDIII-targeting MAb 3H5 and quaternary epitope-targeting MAb 2D22. The MAbs were incubated with immobilized recombinant EDIII or a control antigen (Halo-tag) to deplete EDIII binding Ab and tested for binding to (**B**) EDIII or (**C**) E-dimer (SD).
